# Unique Behavioral and Neurochemical Effects Induced by Repeated Adolescent Consumption of Caffeine-Mixed Alcohol in C57BL/6 Mice

**DOI:** 10.1371/journal.pone.0158189

**Published:** 2016-07-05

**Authors:** Meridith T. Robins, Julie Lu, Richard M. van Rijn

**Affiliations:** 1 Department of Medicinal Chemistry and Molecular Pharmacology, College of Pharmacy, Purdue University, West Lafayette, Indiana, 47907, United States of America; 2 Department of Neuroscience, University of California San Francisco, San Francisco, California, 94158, Unites States of America; University of Leicester, UNITED KINGDOM

## Abstract

The number of highly caffeinated products has increased dramatically in the past few years. Among these products, highly caffeinated energy drinks are the most heavily advertised and purchased, which has resulted in increased incidences of co-consumption of energy drinks with alcohol. Despite the growing number of adolescents and young adults reporting caffeine-mixed alcohol use, knowledge of the potential consequences associated with co-consumption has been limited to survey-based results and in-laboratory human behavioral testing. Here, we investigate the effect of repeated adolescent (post-natal days P35-61) exposure to caffeine-mixed alcohol in C57BL/6 mice on common drug-related behaviors such as locomotor sensitivity, drug reward and cross-sensitivity, and natural reward. To determine changes in neurological activity resulting from adolescent exposure, we monitored changes in expression of the transcription factor ΔFosB in the dopaminergic reward pathway as a sign of long-term increases in neuronal activity. Repeated adolescent exposure to caffeine-mixed alcohol exposure induced significant locomotor sensitization, desensitized cocaine conditioned place preference, decreased cocaine locomotor cross-sensitivity, and increased natural reward consumption. We also observed increased accumulation of ΔFosB in the nucleus accumbens following repeated adolescent caffeine-mixed alcohol exposure compared to alcohol or caffeine alone. Using our exposure model, we found that repeated exposure to caffeine-mixed alcohol during adolescence causes unique behavioral and neurochemical effects not observed in mice exposed to caffeine or alcohol alone. Based on similar findings for different substances of abuse, it is possible that repeated exposure to caffeine-mixed alcohol during adolescence could potentially alter or escalate future substance abuse as means to compensate for these behavioral and neurochemical alterations.

## Introduction

Over the last decade, numerous products containing high levels of caffeine have emerged [1,2]. These products include energy drinks, powdered caffeine, caffeine pills, buccal caffeine pouches, caffeinated peanut butter, and caffeine vaporizer sticks. These highly caffeinated products are disproportionally targeted to adolescents and young adults [3]. Of these products, the most widely used are highly caffeinated energy drinks, which come in a variety of different volumes (from 1.7 oz energy shots to 20 oz. cans) and caffeine concentrations (9–170 mg/oz.) [2,4,5]. Sales of energy drinks grew 60% from 2008 to 2013, illustrating the increased popularity and consumption of these beverages. Yet, increased accessibility of highly caffeinated products has coincided with increased reports of emergency departments visits because of energy drink consumption [6], highlighting the potential harms of exposure to highly caffeinated solutions to adolescents.

While the consumption of large quantities of caffeine itself is problematic [2,7], added health risks arise when caffeine is consumed with alcohol. It has been reported that 23% to 47% of adolescents and young adult alcohol users consume alcohol-mixed energy drinks [8,9]. Surveys of college-aged students suggest this population consumes large amounts of caffeine-mixed alcohol to fulfill hedonistic motives, such as increased pleasure from intoxication and increasing the intensity and/or nature of intoxication [10,11]. However, serious–and sometimes fatal–consequences can occur when mixing caffeine with alcohol [12–14]. While it is clear that consumption of caffeine-mixed alcohol solutions by adolescents and young adults carries a significant acute health risk, the long-term consequences of repeated exposures to caffeine-mixed alcohol are not yet well understood.

The lack of information on the potential long-term risks is particularly concerning given that adolescents, who are the predominant consumers of caffeine-mixed alcohol, are known to be more susceptible to changes in behavioral and neuronal adaptations from exposure to psychostimulants and drugs of abuse than adults [15–17]. Increased responses to cocaine-induced locomotor stimulation and reward have been observed in adolescent mice exposed to caffeine but not in animals exposed to caffeine in adulthood [17], suggesting chronic exposure outcomes in adolescence are not synonymous with exposures outcomes in adulthood. Legal and ethical issues surrounding alcohol use in minors heavily limits caffeine-mixed alcohol studies in human to self-reported survey-based results or in-laboratory performance tasks [18,19]; yet, animal studies provide a viable option for studying the effects of caffeine-mixed alcohol on adolescent behavior in a controlled setting [20]. Importantly, results observed in previous animal studies correlate with reported effects in adolescents and young adults [17,20–22]. Here we developed an animal model using adolescent mice to mimic exposure to caffeine-mixed alcohol as reported by college-aged adults [6,10,11].

Both caffeine and alcohol are known to increase dopamine release in dopaminergic reward pathways, specifically through their actions involving adenosine and dopamine receptors in the dorsal striatum and nucleus accumbens [23,24]. We hypothesized that repeated consumption of caffeine-mixed alcohol causes stronger activation of the dopaminergic reward pathway than caffeine or alcohol alone and could be on par with the levels of dopamine released by commonly abused psychostimulants, such as cocaine, leading to unique behavioral and pharmacological adaptations. To evaluate how chronic adolescent exposure to caffeine-mixed alcohol alters drug-related behaviors, we exposed C57BL/6 mice to caffeine-mixed alcohol throughout adolescence and monitored changes in locomotor sensitivity, ΔFosB accumulation, cocaine preference, cocaine sensitivity, and natural reward to saccharin. We observed unique behavioral and neurochemical effects of repeated caffeine-mixed alcohol exposure in adolescent mice that may indicate that these animals will experience future events involving caffeine-mixed alcohol, natural rewards, or cocaine and/or other psychostimulants differently than animals not exposed to caffeine-mixed alcohol in adolescence.

## Materials and Methods

### Animals

Adolescent (approximately postnatal day 28 [P28]) male and female C57BL/6 mice were obtained from Harlan Inc. (Indianapolis IN, USA) and allowed to acclimate for one week to handling and drug administration before behavioral testing began at postnatal day 35 [25,26]. Unless specified otherwise, mice were grouped housed in single grommet ventilated Plexiglas cages at ambient temperature (21°C) in a room maintained on a reversed 12L:12D cycle (lights off at 10.00, lights on at 22.00) in animal facilities, accredited by the Association for Assessment and Accreditation of Laboratory Animal Care. Food and water were provided ad libitum and mice were not deprived of food or water at any time. All animal procedures were pre-approved by Institutional Animal Care and Use Committees of Purdue University and the University of California San Francisco and conducted in accordance with National Institutes of Health Guide for the Care and Use of Laboratory Animals.

### Drugs and solutions

Caffeine, ethyl alcohol (200 proof), cocaine hydrochloride, and saccharin were obtained from Sigma Aldrich (St. Louis MO, USA). Caffeine (15 mg/kg), alcohol (1.5 g/kg), and caffeine (15 mg/kg) mixed alcohol (1.5 g/kg) solutions were administered via intraperitoneal injection (i.p., diluted in 0.9% saline) or oral gavage (o.g., dissolved in reverse osmosis water). Cocaine (1.5–30 mg/kg, diluted in 0.9% saline) was administered intraperitoneally (i.p.). For transcardial perfusion, a ketamine (Henry Schein Animal Health, Dublin OH, USA) and xylazine (Sigma Aldrich) cocktail of 100:10 mg/kg solution was administered (10 mg/mL i.p.) to induce anesthesia. Phosphate-buffered saline (PBS), 16% paraformaldehyde ampules (Electron Microscopy Sciences, Hatfield PA, USA), and heparin (10 units/mL) (Sigma) were utilized during perfusion. Saccharin solutions were prepared in reverse osmosis water to concentrations of 0.25 mM, 0.5 mM, 1.0 mM, and 2.0 mM.

### Locomotor sensitization via intraperitoneal exposure

**Fig 1:**
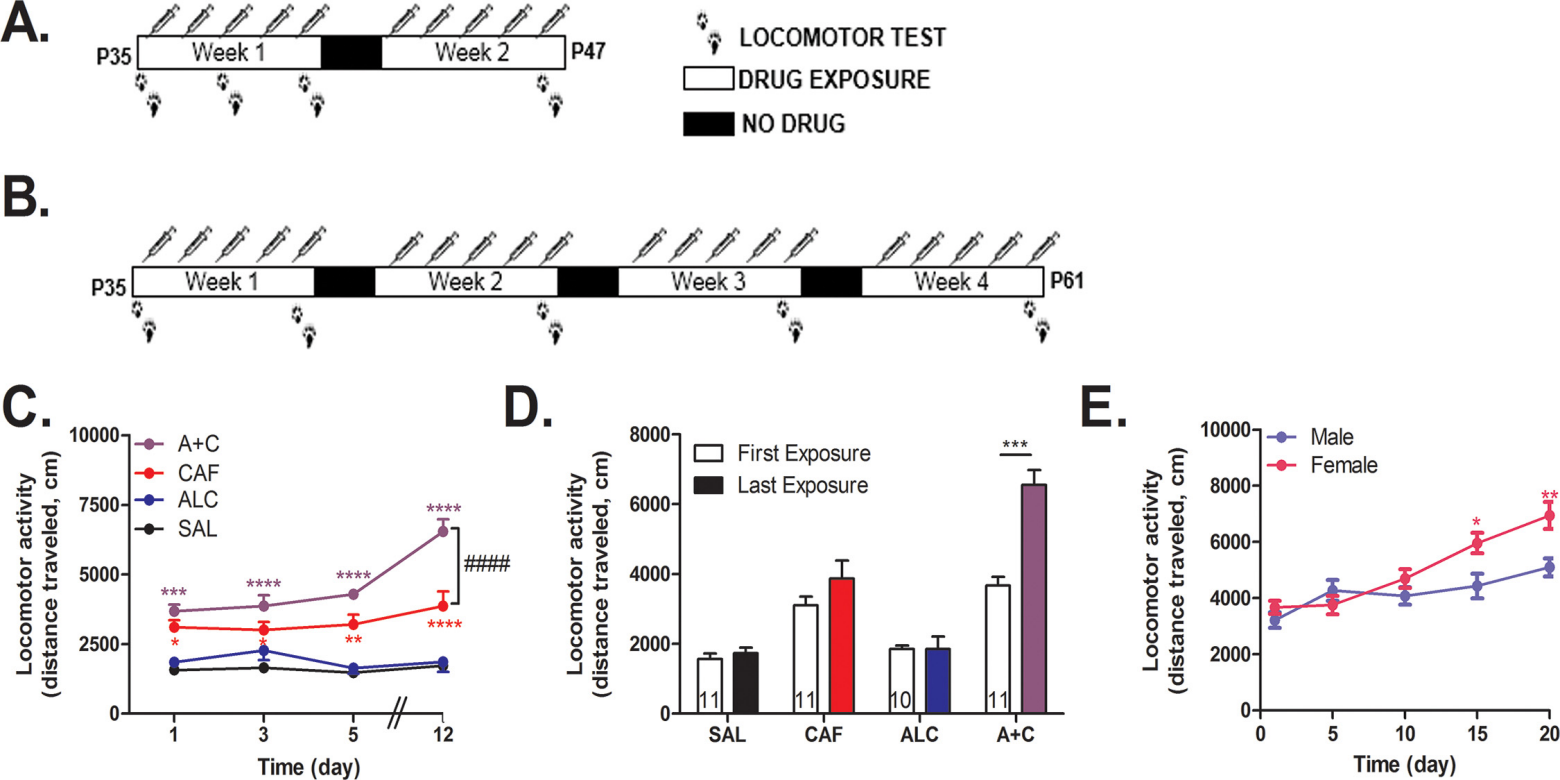
Repeated caffeine-mixed alcohol exposure by intraperitoneal injection during adolescence sensitizes locomotor response with sex specific differences. Adolescent C57BL/6 mice were repeatedly exposed to saline (SAL), 1.5 g/kg alcohol (ALC), 15 mg/kg caffeine (CAF), or caffeine-mixed alcohol (A+C) daily via intraperitoneal injection (n = 9–11 per group) for two weeks (male only, A) of four weeks (male and female, B). Locomotor activity was measured for 60 minutes directly following injection. Total distance traveled per session increased in animals exposed to caffeine-mixed alcohol over the exposure time for adolescent male mice (C). Adolescent male mice exposed to caffeine-mixed alcohol exhibited acute hyperlocomotion and significant locomotor sensitization between first and last exposure session measure in locomotor boxes over two weeks (D). Adolescent female animals sensitized more quickly and robustly than male mice (E) for animals exposed to caffeine-mixed alcohol over four weeks. Statistical significance was assessed by two-way, repeated measures ANOVA (time and treatment) followed by Bonferroni’s Multiple Comparison Test, *, p<0.05; **, p<0.01, ***, p<0.0005, ****, p<0.0001, ####, p<0.0001; data represented as mean ± SEM.

Adolescent male and female C57BL/6 mice (n = 9–11 per group) were administered saline (0.9%), caffeine (15 mg/kg), alcohol (1.5 g/kg), or caffeine-mixed alcohol (15 mg/kg caffeine, 1.5 g/kg alcohol) by intraperitoneal injection for either five days a week for two weeks (male only animals, Fig 1A) or four weeks (male and female animals, Fig 1B). Locomotor activity was measured for 60 minutes in locomotor activity boxes (L 27.3 cm x W 27.3 cm x H 20.3 cm, Med Associates, St Albans City VT, USA) immediately following drug administration on the days depicted in Fig 1A and 1B. Behavioral testing was conducted during the light cycle for each mouse. Mice were habituated to the behavioral testing room one-hour prior to acclimate to fan noise. To reduce the effect of novelty on locomotor activity, mice were habituated to the locomotor boxes the day before the first experiment.

### Locomotor sensitization via oral gavage exposure

**Fig 2:**
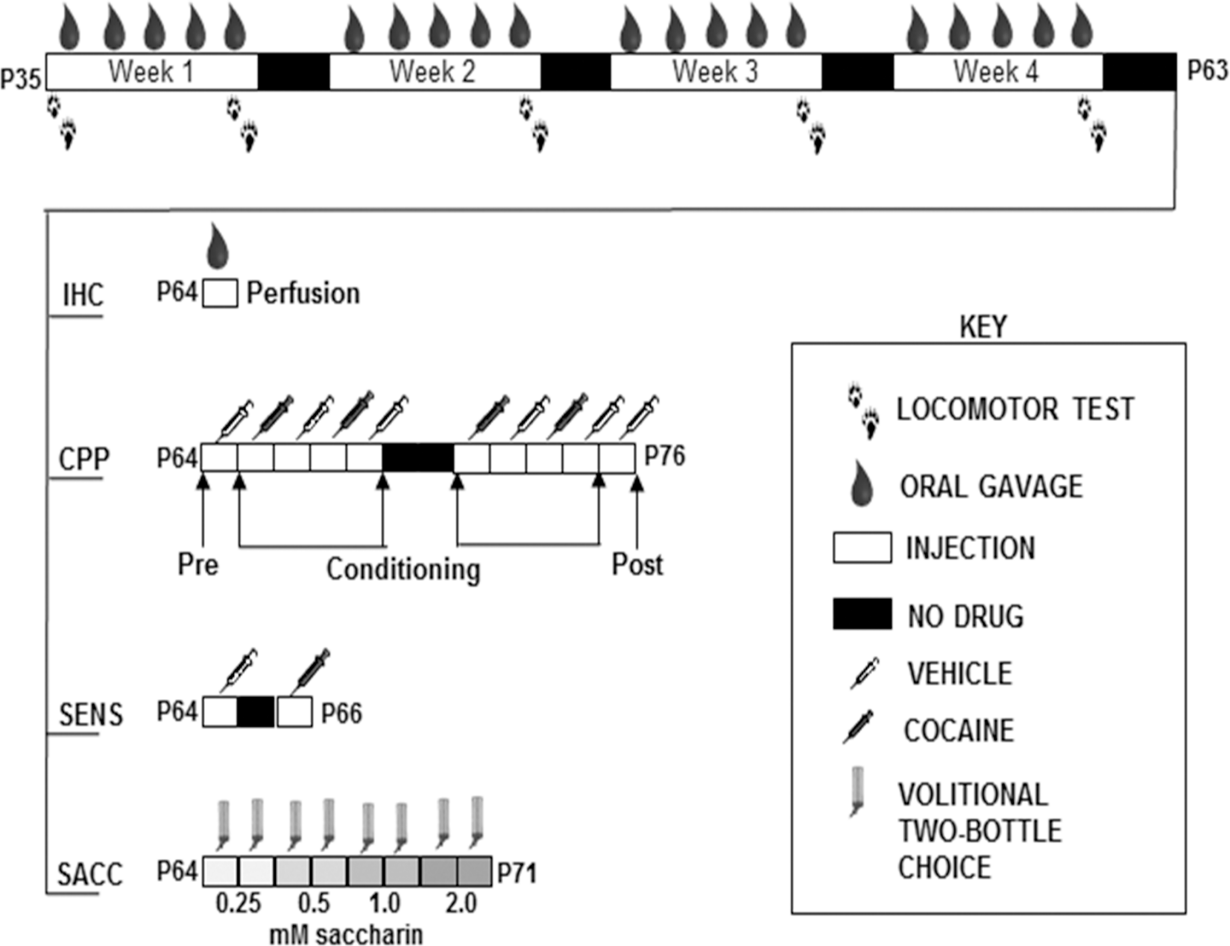
Timeline for adolescent drug exposure via oral gavage for experiments characterizing the effects of caffeine-mixed alcohol on drug related behaviors. Male adolescent C57BL/6 mice were repeatedly exposed to exposed to water (H2O), 1.5 g/kg alcohol (ALC), 15 mg/kg caffeine (CAF) or caffeine-mixed alcohol (A+C), exposure by daily oral gavage (n = 6 per group) for 4 weeks for locomotor monitoring as depicted by the arrows. At the end of four weeks, animals were either perfused after one more drug administration (“IHC”) or subjected to behavioral tasks. Animals under “CPP” were subjected to cocaine conditioned place preference for cross-sensitization to cocaine reward. Animals in “SENS” were monitored for cocaine locomotor cross-sensitization. Natural reward consumption of saccharin was measured in “SACC” through four-hour limited-access, two-bottle choice between concentrations of saccharin (0.25, 0.5, 1.0, and 2.0 mM saccharin) and water for two days at each saccharin concentration.

Adolescent male C57BL/6 mice (n = 6 per group) were administered water, caffeine (15 mg/kg), alcohol (1.5 g/kg), or caffeine-mixed alcohol (15 mg/kg caffeine, 1.5 g/kg alcohol) by oral gavage for five days a week for four weeks (Fig 2). Locomotor activity was measured for 60 minutes in the locomotor activity boxes immediately following drug administration on the days depicted in Fig 2. Behavioral testing was conducted during the active/dark cycle for each mouse. Mice were habituated to the behavioral testing room one-hour prior to acclimate to fan noise. To reduce the effect of novelty on locomotor activity, mice were habituated to the locomotor boxes the day before the first experiment.

### ΔFosB expression levels changes in dorsal striatum and nucleus accumbens

Adolescent male C57BL/6 mice (n = 6 per group) were administered water, caffeine, alcohol, or caffeine-mixed alcohol via oral gavage or cocaine (15 mg/kg, i.p.), five days a week for four weeks during the animal’s dark/active cycle (Fig 2). Three days after the four week period of adolescent exposure, animals were once more exposed to their respective treatment and brains were collected 30 minutes later via transcardial perfusion as previously described by Engle et al, 2013 [27] (Fig 2 “IHC”). Brains were fixed in a 4% paraformaldehyde solution for 24 hours before transfer into 30% sterile sucrose (Sigma) for one week for cryoprotection. Brains were embedded and frozen in Tissue-Tek® O.C.T. compound (VWR, Radnor PA, USA) in tissue molds (VWR) and 50 μm coronal sections were prepared using a cryostat (Leica Microsystems Inc., Buffalo Grove IL, USA). Staining was conducted on free-floating slices for ΔFosB positive cells using primary goat anti-ΔFosB antibody (sc-48-G, Santa Cruz Biotechnology, Dallas TX, USA), diluted 1:1000 and secondary Alexa-Fluor 594 donkey anti-goat antibody (A-11058, Life Technologies, Grand Island NY, USA), diluted 1:1000. Slices were mounted with VectaShield (Vector Laboratories, Burlingame CA, USA) mounting media on microscope slides (Fischer Scientific, Hampton NH, USA), fitted with coverglass (Fischer Scientific), and sealed with nail polish.

Images were acquired via confocal microscopy (Nikon A1) at 20x magnification using an oil immersion objective. Gain and exposure were standardized to slices from a water-treated animal for proper control throughout image capture. For each animal, two images were collected, one image from the left hemisphere and one from the right hemisphere for the brain region of interest. Images were processed using ImageJ software (National Institutes of Health) for the number of ΔFosB positive cells in the dorsal striatum and shell of the nucleus accumbens per image. Positive cells were identified as areas with a specific intensity and area compared to background, as identified through Image J analysis. The total area of analysis for each images = 403072 um^2^.

### Conditioned place preference to cocaine

Adolescent male C57BL/6 mice (n = 8–12 per group) were administered water, caffeine, alcohol, or caffeine-mixed alcohol via oral gavage, five days a week for four weeks as previously described (Fig 2). The following week, mice were conditioned to cocaine in a conditioned place preference paradigm (CPP, Fig 2 “CPP”) [28]. On day 1, mice were injected i.p. with saline and placed in a two-chamber conditioned place preference box (ENV-3013-2, Med Associates) to establish baseline preference the two chambers. Testing chambers contained unique tactile (wired mesh versus metal rod flooring) and visual (horizontal or vertical black and white striped wallpaper) cues for contextual usage to differentiate between the two chambers. Over the following eight conditioning days, mice received daily i.p. injection alternatively with saline or cocaine (1.5, 5, 15, or 30 mg/kg) and were confined for 30 minutes to either a cocaine-paired side or saline-paired side of the box in an unbiased approach. On the final day, saline was administered and the mice were placed in the CPP box in order to freely move between the two boxes for preference testing for 30 minutes (Fig 2). Preference was calculated as the difference in time spent in the cocaine-paired side between the pre- and post-conditioning tests. Mice that spent 70% of time in one side on the pre-conditioning day were excluded from the test. All conditioning was conducted during the dark/active cycle for each mouse.

### Cocaine locomotor cross-sensitization

Adolescent male C57BL/6 mice (n = 7–8 per group) were administered water, caffeine (15 mg/kg), alcohol (1.5 g/kg), or caffeine-mixed alcohol (15 mg/kg caffeine, 1.5 g/kg alcohol) by oral gavage for five days a week for four weeks (Fig 2). Locomotor activity was measured for 60 minutes in the locomotor activity boxes on the first and final day of drug administration. Locomotor activity was measured as described previously for 60 minutes following habituation to the testing room during the animals’ dark/active cycle. Three days after final drug administration, animals were injected with 0.9% saline (i.p.) and placed in the locomotor boxes for baseline locomotor activity for 60 minutes. Two days after this baseline measurement (total of 5 days since last drug treatment), animals were injected with 15 mg/kg cocaine (i.p.) and placed in the locomotor boxes for 60 minutes for total locomotor activity measurement (Fig 2 “SENS”).

### Natural reward to saccharin

Natural reward was monitored through preference of sweet solution (saccharin) versus water in a four-hour, two bottle choice, drinking-in-the-dark paradigm [29] following adolescent exposure to drug solutions. Male adolescent C57BL/6 mice (n = 6–8 per group) were exposed to water or caffeine-mixed alcohol via oral gavage as described previously for four weeks in adolescence, shown in Fig 2. Upon final drug administration during the fourth week, animals were moved into single housing, double grommet cages for fluid consumption monitoring and to allow one weekend of acclimation to new cages. Three days after, saccharin solutions (0.25, 0.5, 1.0, 2.0 mM in reverse osmosis water) were prepared in 50 mL Falcon tubes, fitted with sippers, and distributed to the animals alongside a water control bottle during a four-hour, drinking-in-the-dark period to monitor saccharin consumption preference and volume (Fig 2 “SACC”) [30,31]. Bottles were added two hours into the dark cycle and removed four hours later, allowing behavioral testing during the animals’ active cycle. Weights of the bottles were measured to 0.1 gram. Each concentration was offered to the animals for two consecutive days before moving to the next concentration for total of eight days of drinking. The location of the water and saccharin bottles was reversed between days to prevent habit formation.

### Statistical Analysis

All data are presented as means ± standard error of the mean. The analysis of pharmacological drug effects over time was performed using one-way or two-way, repeated measures ANOVA for adolescent drug treatment and time, followed by a Bonferroni post-hoc test to determine statistically significant differences between groups using GraphPad Prism5 software (GraphPad Software, La Jolla, CA, USA). Student’s unpaired t-test was used for analyzing less than two groups using GraphPad Prism5.

## Results

### Repeated adolescent caffeine-mixed alcohol exposure induces significant locomotor sensitization

We observed that adolescent mice exposed to caffeine-mixed alcohol or caffeine alone by i.p. injection (Fig 1A) displayed significant locomotor activity compared to water or alcohol alone as determined by two-way, repeated measures ANOVA (treatment: F_3, 158_ = 85, *p*<0.0001, time: F_4, 158_ = 7.74, *p*<0.0001), where we also observed a statistically significant interaction effect (interaction time x treatment: F_12, 158_ = 3.22, *p*<0.0004, Fig 1C). Comparison of locomotor activity after the first injection versus the last injection revealed that only caffeine-mixed alcohol exposure caused statistically significant locomotor sensitization (two-way, repeated measures ANOVA for time: F_1, 67_ = 16.70, *p*<0.0001, treatment: F_3, 67_ = 48.50, *p*<0.0001, interaction time x treatment: F_3, 67_ = 8.03, *p*<0.0001, Fig 1D). Female animals sensitized more quickly and robustly than male animals, although this difference was only apparent three weeks into testing (Fig 1B and 1E) as shown by two-way, repeated measures ANOVA for gender: F_1, 17_ = 5.51, *p*<0.0313, time: F_4, 68_ = 23.15, *p*<0.0001, and interaction time x gender: F_4, 68_ = 4.96 *p*<0.0014.

**Fig 3:**
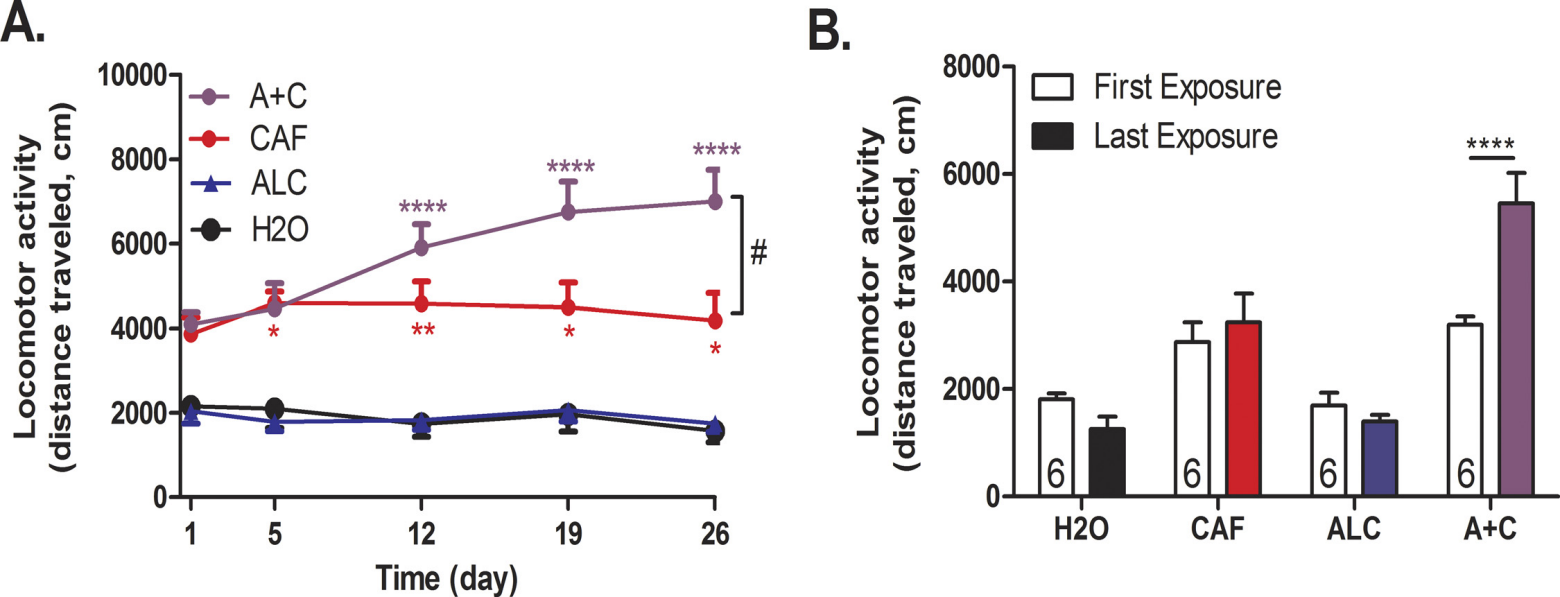
Repeated caffeine-mixed alcohol exposure by oral gavage during adolescence sensitizes locomotor responses. Adolescent C57BL/6 mice were exposed to water (H2O), 1.5 g/kg alcohol (ALC), 15 mg/kg caffeine (CAF) or caffeine-mixed alcohol (A+C), exposure by daily oral gavage (n = 6 per group) for 4 weeks (Fig 2). Locomotor activity was measured for 60 minutes directly following injection. Mice exposed to caffeine-mixed alcohol showed acute hyperlocomotion and significant locomotor sensitization over the course of four weeks (A). Differences in first and last exposure demonstrate the increase in locomotor activity over the locomotor testing sessions (B). Statistical significance was assessed by two-way, repeated measures ANOVA (time and treatment) followed by Bonferroni’s Multiple Comparison Test, *, p<0.05; **, p<0.01, ***, p<0.0005, ****, p<0.0001, #, p<0.05; data represented as mean ± SEM.

In order to increase the physiological relevance of the animal model while maintaining the ability to administer controlled amounts, we changed the exposure route from i.p. to oral gavage (Figs 2 and 3). We found that caffeine and caffeine-mixed alcohol significantly increased locomotor activity over four weeks of exposure (treatment: F_4, 133_ = 66.64, *p<*0.0001, time: F_4, 133_ = 0.67, *p*<0.6117, time x treatment F_16, 133_ = 2.13, *p* = 0.01, Fig 3A). In this model, we again observed that only adolescent mice exposed to caffeine-mixed alcohol showed significant locomotor sensitization versus caffeine alone between first and last drug exposure (two-way, repeated measures ANOVA for time: F_3, 38_ = 3.63, *p* = 0.06, treatment: F_3, 38_ = 35.18, *p*<0.0001, interaction time x treatment: F_3, 38_ = 7.82, *p*<0.0003 Fig 3B), although four weeks of exposure were necessary for these effects to be significantly different from the locomotor activity induced by caffeine alone.

### Animals exposed to caffeine-mixed alcohol in adolescence exhibit significant ΔFosB expression in nucleus accumbens

**Fig 4:**
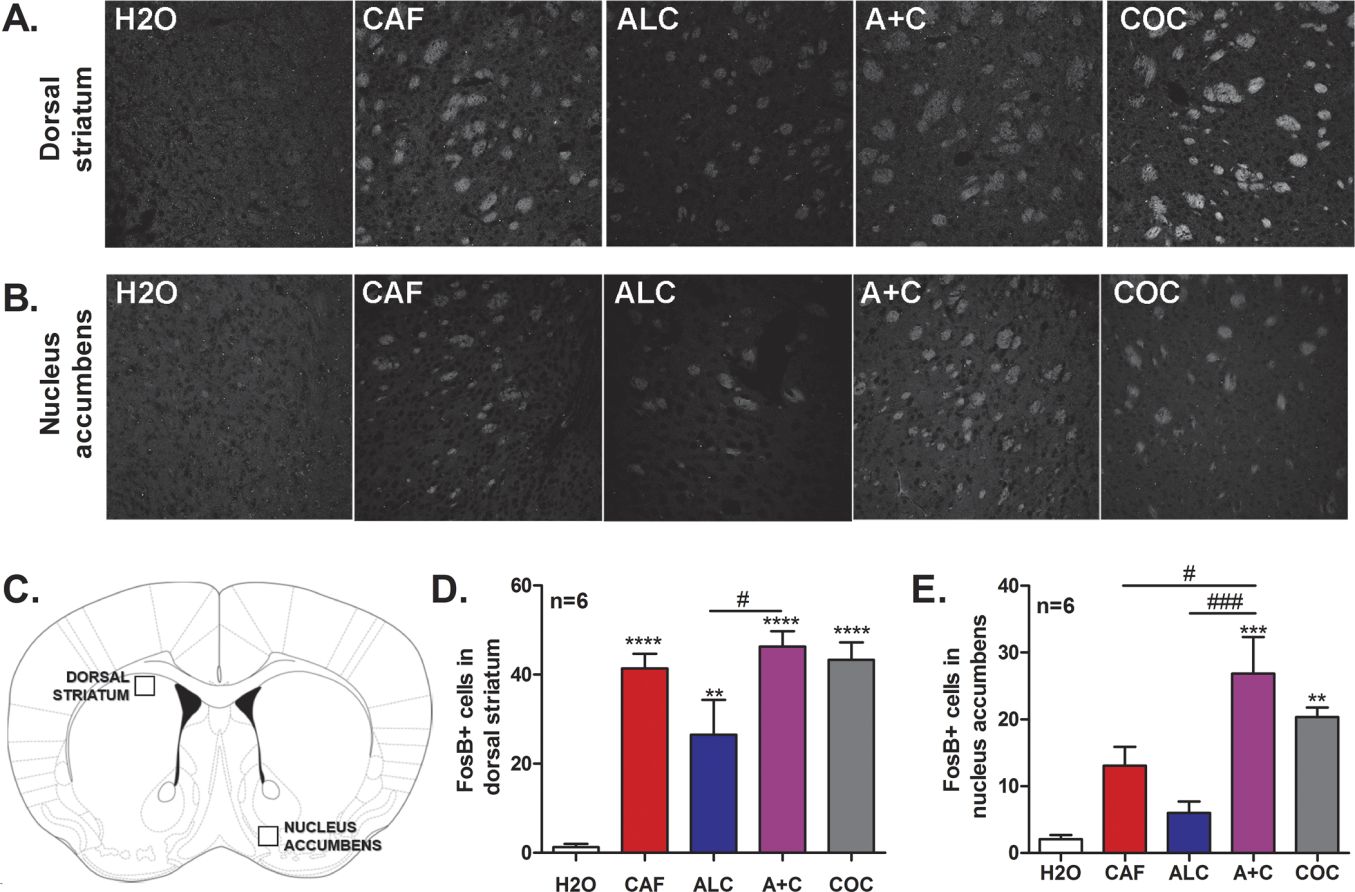
Adolescent exposure to caffeine-mixed alcohol during adolescence significantly increases ΔFosB expression levels in the nucleus accumbens similar to cocaine. Adolescent C57BL/6 mice (n = 6 per group) were repeatedly exposed by oral gavage to water (H2O), 15/mg/kg caffeine (CAF), 1.5 g/kg alcohol (ALC), caffeine-mixed alcohol (A+C), or 15 mg/kg cocaine (i.p., COC) for four weeks in adolescence, as shown in Fig 2. Three days after the final locomotor session, animals were exposed once more to their respective treatment. Brains were removed 30 minutes after exposure to last treatment via transcardial perfusion. Coronal brain slices were immunohistochemically stained for ΔFosB expression in the dorsal striatum (A, D) and nucleus accumbens (B, E), as indicated in C. All treatments increased ΔFosB accumulation in the dorsal striatum compared to water controls (A, D). Increases in ΔFosB accumulation were observed in the nucleus accumbens in animals exposed to caffeine-mixed alcohol compared to alcohol or caffeine alone (B, E). Quantification was achieved by counting the number of ΔFosB for each treatment using ImageJ software. Statistical significance was determined by one-way ANOVA followed by Bonferroni’s Multiple Comparison Test, *, p<0.05; **, p<0.01, ***, p<0.0005, #, p<0.05, ###, p<0.0005; data represented as mean ± SEM.

The locomotor sensitization we observed in adolescent mice exposed to caffeine-mixed alcohol resembled the locomotor sensitization commonly observed upon chronic cocaine exposure [32]. Chronic cocaine exposure is known to induce long-term increases in ΔFosB expression in the mesocortical and nigrostriatal dopaminergic pathways [33], thus we examined whether changes in ΔFosB expression occurred in the dorsal striatum and nucleus accumbens as a result of drug exposure (Fig 2). The shell of the nucleus accumbens was chosen (compared to nucleus accumbens core) as dopamine concentrations are known to preferentially increase in the shell following exposure to drugs of abuse [34]. One-way ANOVA analysis of these data was statistically significant for both dorsal striatum (F_4, 29_ = 17.43, *p<*0.0001, Fig 4A, 4C and 4D) and nucleus accumbens (F_4, 28_ = 10.73, *p*<0.0001, Fig 4B, 4C and 4E) indicating that treatment in general affected ΔFosB expression. Post-hoc analysis with Bonferroni’s multiple comparison test revealed that mice exposed to cocaine, caffeine, alcohol, or caffeine-mixed alcohol exhibited a significant increase in the number of ΔFosB positive cells in the dorsal striatum compared to water controls. Interestingly, mice exposed to caffeine-mixed alcohol or cocaine during adolescence, but not alcohol or caffeine alone, exhibited increased ΔFosB expression in the nucleus accumbens versus water controls.

### Adolescent caffeine-mixed alcohol desensitizes cocaine conditioned place preference

**Fig 5:**
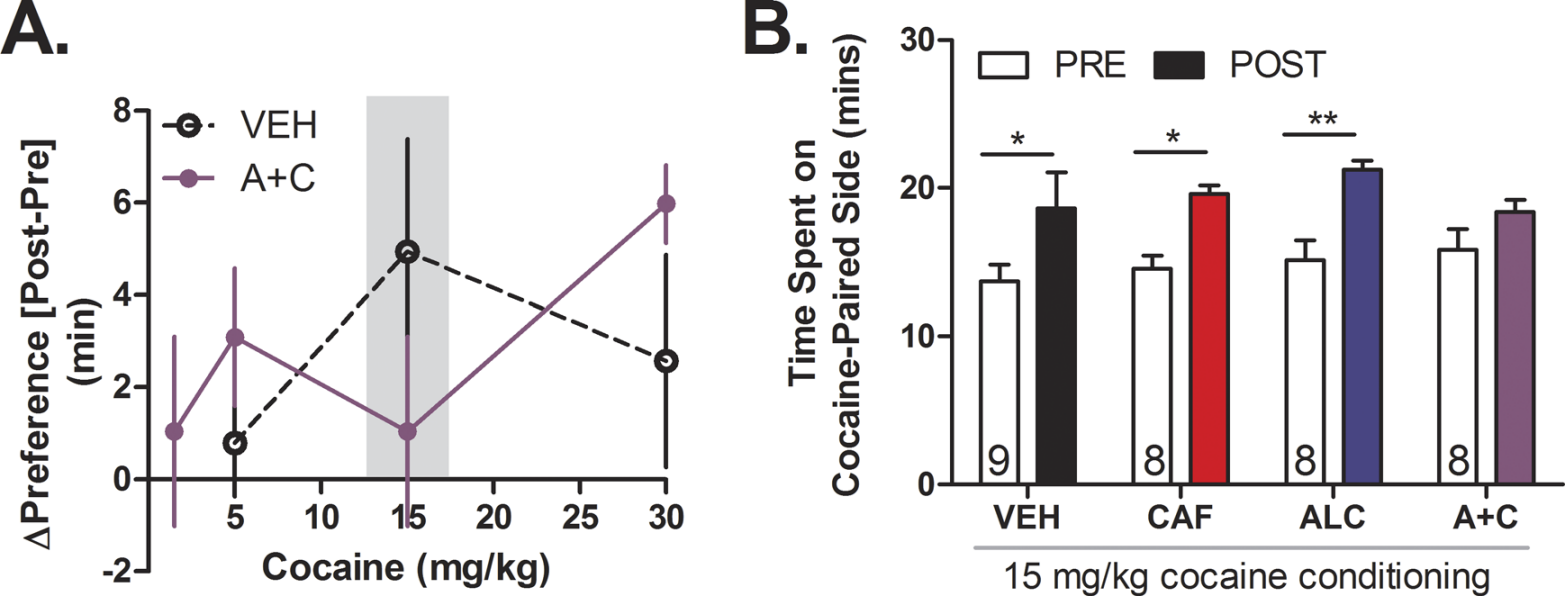
Adolescent exposure to caffeine-mixed alcohol desensitizes cocaine conditioned place preference in early adulthood. Pre-conditioning and post-conditioning time spent on cocaine-paired side for mice treated with water (H2O), 15 mg/kg caffeine (CAF), 1.5 g/kg alcohol (ALC), or caffeine-mixed alcohol (A+C), o.g., for four weeks prior to cocaine conditioning (n = 8–12). Cocaine conditioned began three days after final adolescent drug administration. Cocaine was administered at 1.5, 5, 15, or 30 mg/kg, i.p. doses Cocaine preference, is depicted as the difference in time spent on the cocaine-paired side [change in preference = post-test (minutes)–pre-test (minutes)], (n = 8–12 per group) (A). Animals exposed to water, caffeine, or alcohol alone exhibited conditioned place preference to 15 mg/kg cocaine conditioning (n = 8–11 per group) (B), while this response was attenuated in animals exposed to caffeine-mixed alcohol. Open bars depict pre-conditioning measurement, closed bars depict post-conditioning measurement. Significance by two-way, repeated measures ANOVA with Bonferroni’s Multiple Comparisons Test, *, p<0.05; **, p<0.01; data represented as mean ± SEM.

Considering the similarities between caffeine-mixed alcohol and cocaine with regard to locomotor sensitization, ΔFosB expression, and previous reports of caffeine induced sensitization of cocaine place preference [17,32], we next tested whether adolescent mice exposed to caffeine-mixed alcohol would show altered sensitivity to the rewarding properties of cocaine [32,33]. Mice were exposed to daily oral gavage injections of water, caffeine (15 mg/kg), alcohol (1.5 g/kg) or caffeine-mixed alcohol for four weeks during adolescence. Three days after final drug exposure, animals were subjected to cocaine conditioned place preference (Fig 2). Dose of 1.5, 5, 15, and 30 mg/kg were used to test preference exposed to caffeine-mixed alcohol in adolescence in separate cohorts of animals. Whereas animals exposed to water exhibited the strongest cocaine place preference to a dose of 15 mg/kg (Fig 5A) in accordance with that previously reported Hnasko et al., 2007 [35], caffeine-mixed alcohol exposed mice only showed significant place preference at 30 mg/kg of cocaine (two-way, repeated measures ANOVA for time: F_1,13_ = 13.47, *p* = 0.0023, treatment: F_1,13_ = 0.90, *p* = 0.3600, interaction time x treatment: F_1,13_ = 2.14, *p* = 0.1668, S1C Fig). No cocaine conditioned place preference was observed at 1.5 mg/kg for animals exposed to caffeine-mixed alcohol (S1A Fig) and no conditioning was observed in caffeine-mixed alcohol or water animals at 5 mg/kg cocaine (two-way, repeated measures ANOVA for time: F_1,16_ = 4.36, *p* = 0.053, treatment: F_1,16_ = 0.04, *p* = 0.8402, interaction time x treatment: F_1,16_ = 1.54, *p* = 0.2320, S1B Fig). Cocaine induced place preference at a dose of 15 mg/kg cocaine across all treatment groups except caffeine-mixed alcohol exposed animals, indicating that only caffeine-mixed alcohol exposed mice displayed desensitized place preference (two-way, repeated measures ANOVA for time: F_1,29_ = 28.17, *p*<0.0001, treatment: F_3,29_ = 0.70, *p*<0.5600, interaction time x treatment: F_3,29_ = 0.72, *p*<0.5501, Fig 5B).

We observed no difference in cocaine induced hyperlocomotion between water and caffeine-mixed alcohol exposed animals upon their first cocaine exposure during conditioning at any of the tested cocaine conditioning doses (S2A Fig). Additionally, there were no differences in 15 mg/kg cocaine induced locomotor activity during first conditioning session to cocaine between adolescent treatment groups (S2B Fig), suggesting that the attenuation in place preference observed in animals exposed to caffeine-mixed alcohol was not a result of alterations in locomotor response to cocaine. Adolescent exposure to caffeine-mixed alcohol also did not impact general locomotor activity during the pre-conditioning test day compared to water controls, although Bonferroni’s post-hoc analysis did show that caffeine exposed mice had significantly more locomotor activity than animals exposed to alcohol in adolescence (one-way ANOVA F_3,29_ = 4.976, *p* = 0.004, S2C Fig).

### Repeated exposure to caffeine-mixed alcohol attenuates the sensitizing effects of caffeine alone to cocaine locomotor cross-sensitization

**Fig 6:**
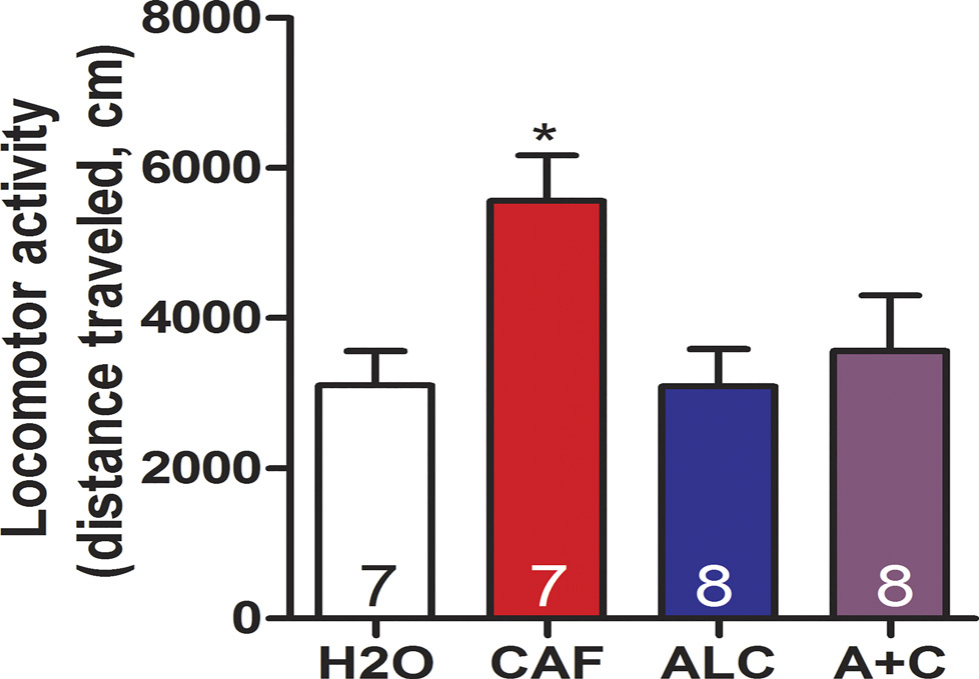
Exposure to caffeine-mixed alcohol attenuates caffeine-induced cocaine locomotor cross-sensitivity. Adolescent male animals exposured to water (H2O), 15 mg/kg caffeine (CAF), 1.5 g/kg alcohol (ALC), or caffeine-mixed alcohol (15 mg/kg caffeine, 1.5 g/kg alcohol, A+C) were challenged to 15 mg/kg cocaine in adulthood (n = 7–8 per group). Animals repeatedly exposed to caffeine alone exhibited increased cocaine locomotor cross-sensitization than animals exposed to water, alcohol, or caffeine-mixed alcohol. Significance by one-way ANOVA, *, p<0.05; data represented as mean ± SEM.

To investigate the effects of caffeine-mixed alcohol on cocaine locomotor cross-sensitivity, animals were exposed to 15 mg/kg cocaine after adolescent treatment (Fig 2). Exposure to caffeine alone increased both baseline (S3 Fig) and cocaine-induced increases in ambulation after adolescent treatment (Fig 6), while exposure to water, alcohol, or caffeine-mixed alcohol did not (one-way ANOVA for baseline: F_3,29_ = 5.556, *p* = 0.0044, cocaine: F_3,29_ = 3.723, *p* = 0.0237).

### Caffeine-mixed alcohol exposure increases natural reward consumption and preference

**Fig 7:**
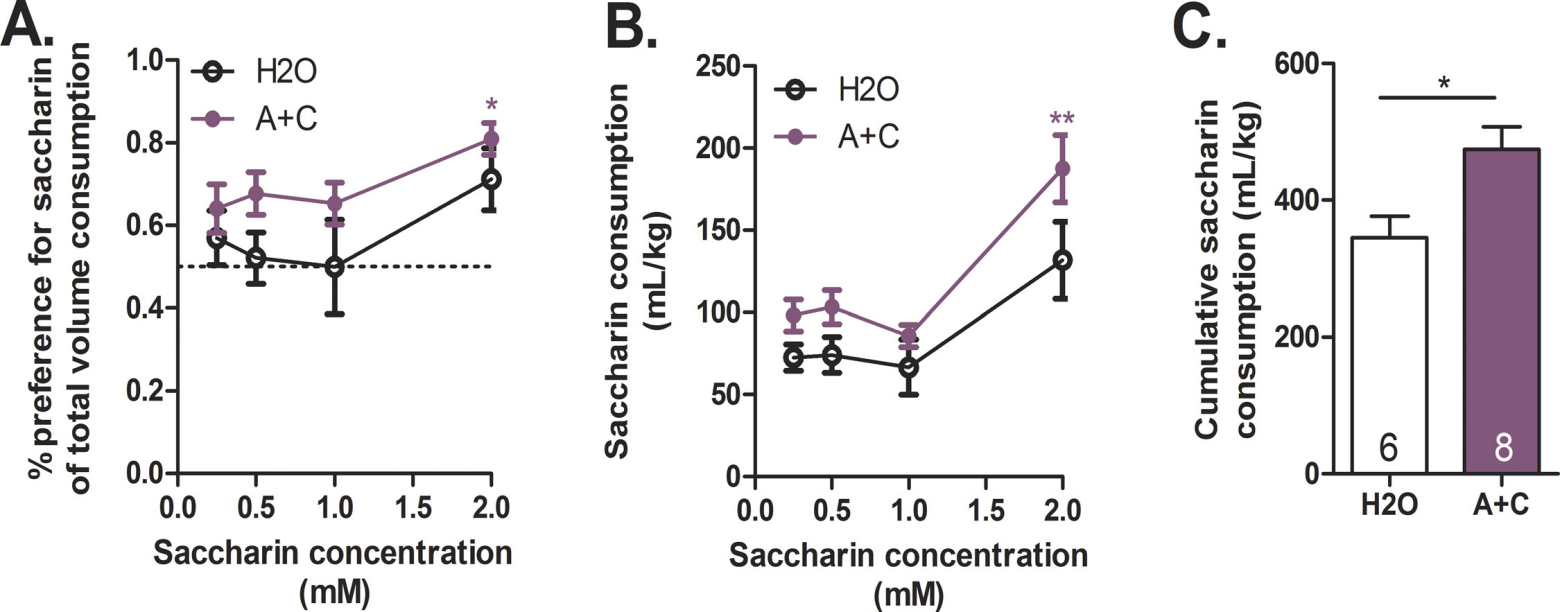
Caffeine-mixed alcohol exposure in adolescence increases natural reward consumption and preference. Adolescent exposure to caffeine-mixed alcohol (15 mg/kg caffeine, 1.5 g/kg alcohol, A+C) or water (H2O) altered natural reward consumption and preference in adulthood (n = 6–8 per group). An increase in saccharin preference was observed throughout testing between animals exposed to caffeine-mixed alcohol versus water controls (A). Animals exposed to caffeine-mixed alcohol consumed significantly more 2.0 mM saccharin solution (B) with a greater saccharin solution consumption overall compared to water controls (C). Significance by two-way ANOVA, *, p<0.05; **, p<0.01 or unpaired t-test, *, p<0.05; data represented as mean ± SEM.

We next investigated if exposure to caffeine-mixed alcohol during adolescence altered natural reward consumption and preference [30]. To prevent satiation, saccharin solutions were chosen because of saccharin’s lack of caloric value compared to sucrose, which could inhibit drinking during the four-hour access period. Animals exposed to caffeine-mixed alcohol (15 mg/kg caffeine, 1.5 g/kg alcohol) during adolescence increased saccharin solution preference compared to animals exposed to water as observed by two-way, repeated measures ANOVA for adolescent treatment: F_1,12_ = 5.95, *p* = 0.031, saccharin concentration: F_3,36_ = 3.59, *p* = 0.023 (Fig 7A). Two-way, repeated measures ANOVA revealed significant differences in saccharin consumption as well, with Bonferroni post-hoc analysis indicating that animals exposed to caffeine-mixed alcohol consumed significantly larger quantities of 2 mM saccharin (Fig 7B, treatment: F_1,12_ = 7.62, *p* = 0.017, saccharin concentration: F_3,36_ = 16.13, *p*<0.0001). Analysis of cumulative saccharin intake revealed the same significant effect as (area under the curve shown in Fig 7C) as analyzed by student’s t-test.

## Discussion

To study the effect of adolescent caffeine-mixed alcohol exposure on drug-related behaviors, we developed a mouse model that enabled us to observe several unique features resulting from repeated adolescent exposure to caffeine-mixed alcohol compared to caffeine or alcohol alone. We exposed animals to caffeine-mixed alcohol (15 mg/kg caffeine, 1.5 g/kg alcohol) by intraperitoneal and oral gavage administrations throughout the span of mouse adolescence [post-natal days 30–60] [36,37]. The alcohol dose of 1.5 g/kg was chosen as it is high enough to induce intoxication without inducing severe locomotor impairment and C57BL/6 mice routinely reach BEC of >0.08 mg/ml [38]. The caffeine dose chosen provided clear stimulation in C57BL/6 mice as evident from increased locomotor activity (Figs 1 and 3). The exposure model utilized mirrors patterns and levels of repeated binge consumption of caffeine-mixed alcohol self-reported by adolescents and young adults [11]. Our results, obtained by monitoring changes in locomotor activity, ΔFosB accumulation, and natural and drug reward sensitivity, support the emerging idea that repeated exposure to caffeine-mixed alcohol poses a risk to adolescent behavioral and neurological development.

Previous studies have shown that the addition of alcohol (1.75–3.25 g/kg) to caffeine (15 mg/kg) can acutely enhance the locomotor effects induced by caffeine alone [21] and locomotor sensitization observed upon repeated exposures of caffeine-mixed alcohol (15 mg/kg + 4 g/kg) [22]. In our animal model using the same dose of caffeine (15 mg/kg), we observed significant locomotor sensitization at lower doses of alcohol (1.5 g/kg) than previously observed (4 g/kg) [22]. This effect was retained for both intraperitoneal and oral gavage drug administration, the latter method being a more relevant route of administration for proper comparison to human consumption and metabolism (Figs 1–3). The increased locomotor activity upon adolescent exposure to caffeine-mixed alcohol was in accordance with previous data [20–22] showing that mixing caffeine with alcohol may diminish the sedative properties of alcohol through caffeine’s stimulant properties, giving rise to a “wide-awake drunk” behavioral state [20]. Additionally, we observed sex differences in response to repeated caffeine-mixed alcohol exposure, with female adolescent animals sensitizing more quickly and robustly than male mice (Fig 1). Our results suggest that repeated caffeine-mixed alcohol consumption in females may be more problematic than repeated exposures in male, aged-matched counterparts. The similarity in increased locomotor sensitivity observed with caffeine-mixed alcohol in females is in accordance with that observed for other psychostimulants [39,40], such as cocaine, suggesting that activation of similar brain regions may occur upon repeated administration of caffeine-mixed alcohol and common psychostimulants.

Neurons in the nucleus accumbens and dorsal striatum that are exposed for a prolonged period to high concentrations of dopamine, e.g. by repeated cocaine exposure, are known to increase expression of ΔFosB, a transcription factor that accumulates upon chronic drug exposure [32,33,41]. Both caffeine and alcohol increase dopamine levels in the mesolimbic and nigrostriatal dopamine systems by affecting firing of dopaminergic neurons [23,42,43], and increased dopaminergic tone in the dorsal striatum has been correlated with enhanced locomotor activity [44]. Therefore, we hypothesized that if caffeine-mixed alcohol-induced-locomotor sensitization was attributed to additive or synergistic striatal release of dopamine, we would observe increased ΔFosB expression in the dorsal striatum and/or nucleus accumbens. We observed that adolescent exposure to caffeine, caffeine-mixed alcohol, and cocaine significantly increased ΔFosB expression in the dorsal striatum compared to water controls (Fig 4). More importantly, significant increases in ΔFosB expression were observed in the nucleus accumbens in animals exposed to caffeine-mixed alcohol or cocaine, but not mice exposed to alcohol or caffeine alone (Fig 4), supporting our hypothesis that caffeine-mixed alcohol can induce stronger dopamine release than caffeine or alcohol exposure alone. Previous studies have observed statistically significant alcohol-induced ΔFosB expression in the nucleus accumbens, but this increase was observed at concentrations much higher than used in our experiment (8–12 g/kg vs 1.5 g/kg) [41], further suggesting that the combination of caffeine-mixed alcohol induces ΔFosB accumulation at lower levels of alcohol intoxication than those previously reported. Our observation that only mice exposed to caffeine-mixed alcohol and not caffeine alone display increased accumbal ΔFosB expression and heightened locomotor sensitization is in agreement with reports showing that cocaine directly injected in the nucleus accumbens induces locomotor sensitization [45–47], emphasizing the role of increased dopamine levels in the nucleus accumbens and increased locomotor stimulation.

The nucleus accumbens is heavily involved in reward-associated learning and behaviors, specifically to drugs of abuse, while the dorsal striatum is involved in decision-making, habitual action, and response control [48]. Enhanced ΔFosB expression in the nucleus accumbens has been previously correlated with increased locomotor sensitization, increased cocaine reward [32], as well as increased place preference to other non-stimulant drugs of abuse, such as morphine [49]. As our mice exposed to repeated caffeine-mixed alcohol exhibited locomotor sensitization and increased accumbal ΔFosB expression, we hypothesized that these mice would also exhibit enhanced cocaine conditioned place preference compared to animals exposed to caffeine or alcohol alone [32,33].

Against our initial hypothesis, animals exposed to caffeine-mixed alcohol exhibited attenuated cocaine place preference to 15 mg/kg cocaine. Instead, caffeine-mixed alcohol exposed animals exhibited “equi-rewarding” effects to 30 mg/kg cocaine when compared to 15 mg/kg cocaine reward for animals exposed to water, caffeine, or alcohol alone (Fig 5, S1 Fig). One possible interpretation of our data is that repeated exposure to caffeine-mixed alcohol caused stronger dopamine release than exposure to caffeine or alcohol alone and potentially desensitized subsequent cocaine reward responses. This hypothesis is in line with our observation that cocaine place preference was only desensitized in mice exposed to caffeine-mixed alcohol, but not caffeine or alcohol.

If exposure to repeated caffeine-mixed alcohol during adolescence caused desensitization of the dopamine system in young adults, we hypothesized that mice repeatedly exposed to caffeine-mixed alcohol would show limited cocaine cross-sensitization, which has been reported for previous exposure to alcohol and caffeine [17,50]. Indeed, whereas adolescent exposure to 15 mg/kg caffeine sensitized locomotor responses to cocaine as previously reported [17], mice exposed to caffeine-mixed alcohol or 1.5 g/kg alcohol did no show cocaine locomotor cross-sensitization (for alcohol alone, this effect may result from the lower cocaine and alcohol doses utilized compared to those previously reported by Itzhak and Martin, 1999) (Fig 6). Several studies have shown that a decrease in drug reward found in conditioned place preference can be associated with increased self-administration, because animals need to administer more of the drug to obtain the same reward or stimulatory effect [51–56]. Thus, our results could indicate that mice exposed to repeated caffeine-mixed alcohol during adolescence may be at greater risk for future abuse of rewarding substances. To test this hypothesis, we investigated how repeated exposure to caffeine-mixed alcohol in adolescence would alter intake of a natural reward (saccharin) in adulthood [30,31,57,58]. In support of our hypothesis, we found that mice exposed to caffeine-mixed alcohol increased voluntary saccharin consumption and preference compared to water control mice (Fig 7). Importantly, animals exposed to caffeine-mixed alcohol did not display an anhedonic response, as animals continued to consume saccharin at levels higher than control animals suggesting that caffeine-mixed alcohol exposure did not decrease reward-seeking motivation.

It is important to note that our adolescent locomotor and ΔFosB measurements were conducted in animals directly after drug administration, while the rest of our behavioral data was collected from animals at a minimum of three days after final adolescent drug exposure. Abstinence and possible withdrawal from the adolescent drug treatments may explain why the increase in locomotor sensitization and ΔFosB expression did not correlate with increased drug reward and sensitivity [32,33,59]. Yet, with attenuated cocaine preference, decreased cocaine locomotor sensitivity, and increased natural reward consumption in adulthood, our results continue to suggest that repeated caffeine-mixed alcohol in adolescence alters reward and response threshold to psychostimulants and natural rewards via desensitization of dopamine reward pathways. Previously, animals characterized as low quinpirole (selective dopamine D_2_ receptor agonist) responders exhibited decreased cocaine conditioned place preference compared to animals with high quinpirole response, potentially as a result of decreased dopamine D_2_ receptor levels in the brain [60]. Dopamine D_2_ receptors are known to undergo receptor downregulation and degradation upon stimulation [61,62]. Thus it is possible that repeated exposure to caffeine-mixed alcohol increased dopamine release by such extent (i.e. more so than alcohol or caffeine alone can accomplish) that it caused desensitized/downregulation of D_2_ receptors [61,63,64]. We hypothesize that additional measurements on drug self-administration to drugs of abuse, such as cocaine or other psychostimulants, would observe an escalation in drug administration resulting from this desensitized response and reward threshold alteration.

The persistent marketing of highly caffeinated products will increase the likelihood of adolescent exposure to highly caffeinated alcoholic beverages, thus understanding the developmental risks of caffeine-mixed alcohol consumption on adolescent behavior and drug reward is vital. Here, we developed a physiologically relevant animal model to investigate the effects of repeated caffeine-mixed alcohol exposure during adolescence for alterations in drug-related behaviors and neuronal activation of the dopamine reward systems. From our model, we observed that repeated adolescent exposure to caffeine-mixed alcohol induced locomotor sensitization, increased expression of transcription factors related with chronic neuronal activation, and altered cocaine conditioned place preference. A desensitized response to cocaine preference and locomotor cross-sensitization was observed in animals exposed to caffeine-mixed alcohol, suggesting a desensitized dopamine reward system, which was supported by increased natural reward consumption and preference to saccharin solutions. Our data provides *in vivo* evidence that highlights several potential health risks associated with repeated exposure to caffeine-mixed alcohol. How these results compare with future drug taking events is currently unknown, but our results suggest that repeated caffeine-mixed alcohol consumption may lead to increased reward thresholds for natural and drug-related rewards, leading to an escalation in reward consumption to reach that threshold. Combined with human data reporting the dangers of acute adolescent consumption of caffeine-mixed alcohol [6,11,65], our results should open up a dialogue about the potential safety risks and marketing strategies of highly caffeinated products to adolescents and young adults.

## Supporting Information

S1 Fig Adolescent mice exposed to caffeine-mixed alcohol display cocaine conditioned place preference at high dose of 30 mg/kg cocaine, but not 1.5 or 5 mg/kg cocaine. Adolescent C57BL/6 mice exposed to caffeine-mixed alcohol (15 mg/kg caffeine, 1.5 g/kg alcohol, A+C) did not display conditioned place preference to 1.5 mg/kg cocaine (n = 12) (A). Caffeine-mixed alcohol nor water (H2O) exposed animals displayed conditioned place preference to 5 mg/kg cocaine (n = 8–10) (B). At 30 mg/kg, animals exposed to caffeine-mixed alcohol spent more time on the drug paired side after drug conditioning, while animals exposed to water did not (n = 7–8) (C). Open bars depict pre-conditioning measurement, closed bars depict post-conditioning measurement. Significance by two-way, repeated measures ANOVA, **, p<0.01 or unpaired t-test, *, p<0.05; data represented as mean ± SEM.
(TIF)

S2 Fig Attenuation of cocaine CPP in caffeine-mixed alcohol exposed mice is not due to differences in cocaine-induced psychostimulation, or changes in general locomotor activity. Dose-dependent cocaine induced psychostimulation is equal between water (H2O) and caffeine-mixed alcohol (15 mg/kg caffeine, 1.5 g/kg alcohol, A+C) exposed adolescent C57BL/6 mice (n = 8–12). No alterations in locomotor activity were observed in animals exposed to caffeine-mixed alcohol compared to water controls as tested cocaine conditioning doses (A). No significant difference was observed upon first cocaine conditioning session to 15 mg/kg cocaine between adolescent treatment groups (n = 8–11) (B). Pretest locomotor activity between adolescent treatments of water, caffeine (15 mg/kg), alcohol (1.5 g/kg), or caffeine-mixed alcohol were observed between animals exposed to caffeine versus alcohol groups (C). Significance by two-way, repeated measures ANOVA, **, p<0.01; data represented as mean ± SEM.
(TIF)

S3 Fig Exposure to caffeine increases basal locomotor activity in adulthood. Adolescent male animals exposured to water (H2O), 15 mg/kg caffeine (CAF), 1.5 g/kg alcohol (ALC), or caffeine-mixed alcohol (15 mg/kg caffeine, 1.5 g/kg alcohol, A+C) were challenged to 15 mg/kg cocaine in adulthood (n = 7–8 per group). Animals repeatedly exposed to caffeine alone exhibited increased baseline locomotor activity than animals exposed to water, alcohol, or caffeine-mixed alcohol. Significance by one-way ANOVA, **, p<0.01; data represented as mean ± SEM.
(TIF)
