# Supplementary figures and images for: Unique Behavioral and Neurochemical Effects Induced by Repeated Adolescent Consumption of Caffeine-Mixed Alcohol in C57BL/6 Mice
Source: PLoS One. 2016 Jul 5;11(7):e0158189. doi: 10.1371/journal.pone.0158189 (PMC4933367; doi:10.1371/journal.pone.0158189)

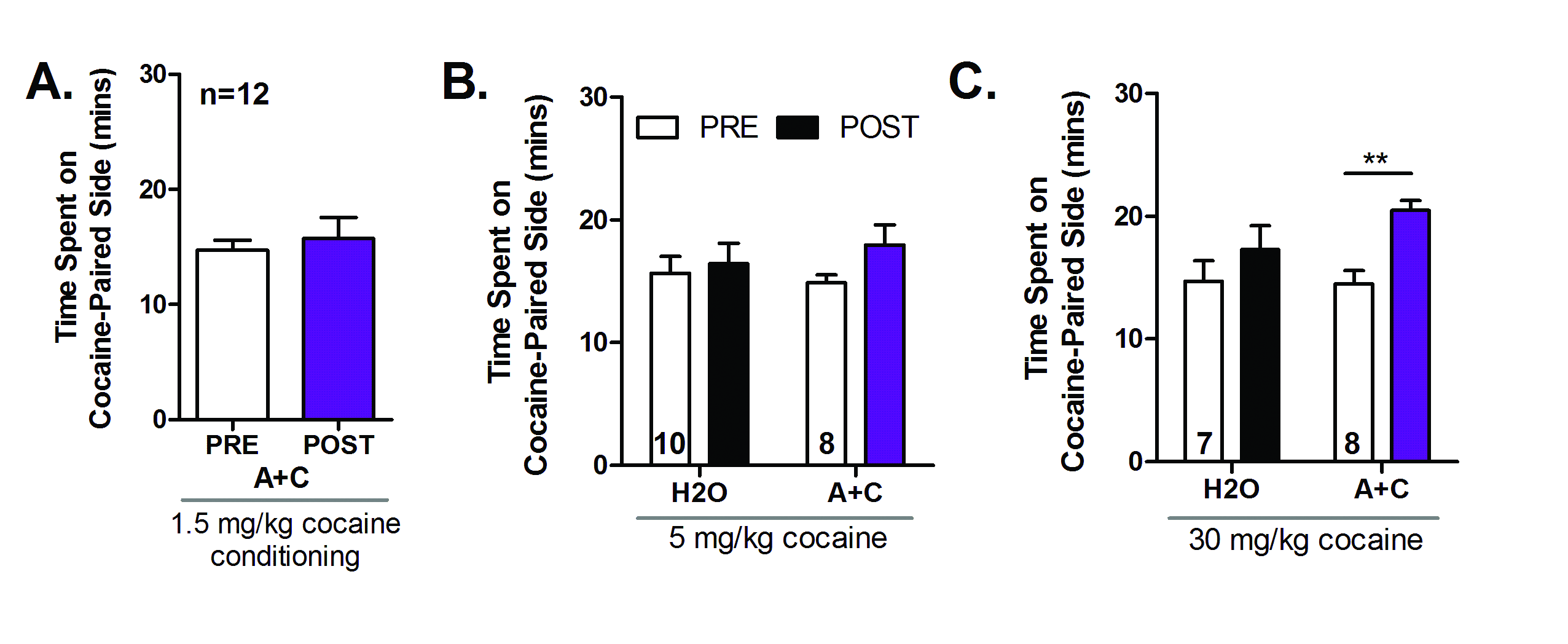

Supplement: S1 Fig — Adolescent C57BL/6 mice exposed to caffeine-mixed alcohol (15 mg/kg caffeine, 1.5 g/kg alcohol, A+C) did not display conditioned place preference to 1.5 mg/kg cocaine (n = 12) (A). Caffeine-mixed alcohol nor water (H2O) exposed animals displayed conditioned place preference to 5 mg/kg cocaine (n = 8–10) (B). At 30 mg/kg, animals exposed to caffeine-mixed alcohol spent more time on the drug paired side after drug conditioning, while animals exposed to water did not (n = 7–8) (C). Open bars depict pre-conditioning measurement, closed bars depict post-conditioning measurement. Significance by two-way, repeated measures ANOVA, **, p<0.01 or unpaired t-test, *, p<0.05; data represented as mean ± SEM. (TIF) [file pone.0158189.s001.tif]

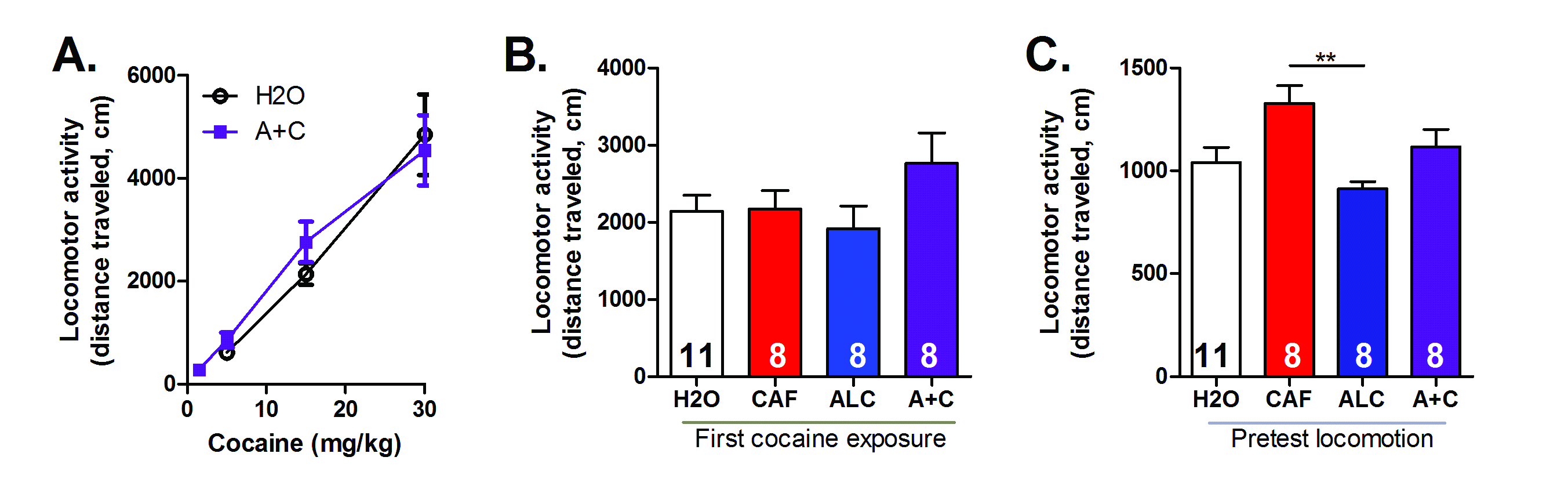

Supplement: S2 Fig — Dose-dependent cocaine induced psychostimulation is equal between water (H2O) and caffeine-mixed alcohol (15 mg/kg caffeine, 1.5 g/kg alcohol, A+C) exposed adolescent C57BL/6 mice (n = 8–12). No alterations in locomotor activity were observed in animals exposed to caffeine-mixed alcohol compared to water controls as tested cocaine conditioning doses (A). No significant difference was observed upon first cocaine conditioning session to 15 mg/kg cocaine between adolescent treatment groups (n = 8–11) (B). Pretest locomotor activity between adolescent treatments of water, caffeine (15 mg/kg), alcohol (1.5 g/kg), or caffeine-mixed alcohol were observed between animals exposed to caffeine versus alcohol groups (C). Significance by two-way, repeated measures ANOVA, **, p<0.01; data represented as mean ± SEM. (TIF) [file pone.0158189.s002.tif]

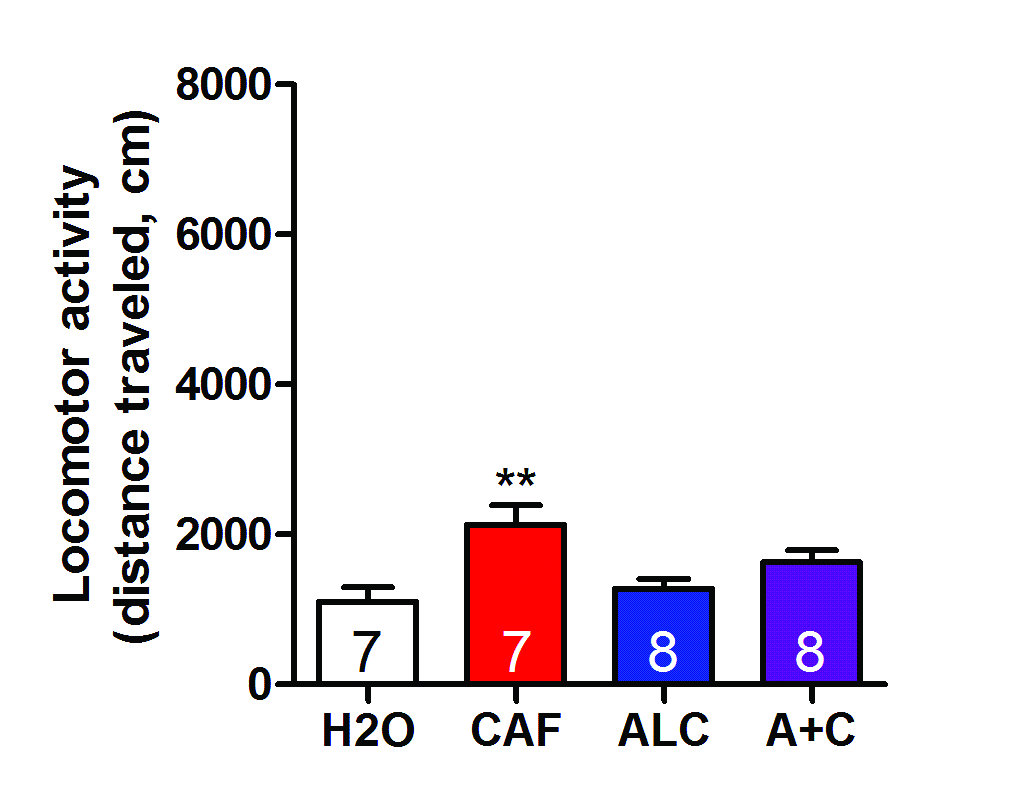

Supplement: S3 Fig — Adolescent male animals exposured to water (H2O), 15 mg/kg caffeine (CAF), 1.5 g/kg alcohol (ALC), or caffeine-mixed alcohol (15 mg/kg caffeine, 1.5 g/kg alcohol, A+C) were challenged to 15 mg/kg cocaine in adulthood (n = 7–8 per group). Animals repeatedly exposed to caffeine alone exhibited increased baseline locomotor activity than animals exposed to water, alcohol, or caffeine-mixed alcohol. Significance by one-way ANOVA, **, p<0.01; data represented as mean ± SEM. (TIF) [file pone.0158189.s003.tif]
